# Video-Games Do Not Negatively Impact Adolescent Academic Performance in Science, Mathematics or Reading

**DOI:** 10.1371/journal.pone.0087943

**Published:** 2014-04-03

**Authors:** Aaron Drummond, James D. Sauer

**Affiliations:** 1 School of Education, Flinders University, Adelaide, South Australia, Australia; 2 School of Psychology, University of Portsmouth, Portsmouth, United Kingdom; Cardiff University, United Kingdom

## Abstract

Video-gaming is a common pastime among adolescents, particularly adolescent males in industrialized nations. Despite widespread suggestions that video-gaming negatively affects academic achievement, the evidence is inconclusive. We reanalyzed data from over 192,000 students in 22 countries involved in the 2009 Programme for International Student Assessment (PISA) to estimate the true effect size of frequency of videogame use on adolescent academic achievement in science, mathematics and reading. Contrary to claims that increased video-gaming can impair academic performance, differences in academic performance were negligible across the relative frequencies of videogame use. Videogame use had little impact on adolescent academic achievement.

## Introduction

Video-gaming is common among adolescents in industrialized countries, with prevalence rates higher than 75% [1]. Could this common pastime negatively influence adolescents' academic performance? The exciting, fast-paced nature of many videogames could conceivably compromise children's ability to focus on less attention-grabbing tasks (e.g., schoolwork). Consistent with this idea, increased video-gaming has been associated with (a) higher rates of teacher-reports of student attention problems [2] and (b) poorer sleep efficiency [3]. Attentional deficits and poor sleep could both plausibly impair academic performance. Further, increased time spent video-gaming may also reduce home study time and, potentially, academic performance [4].

Presently, evidence on whether video-gaming negatively affects academic achievement is too weak for causal claims [4]. Although some researchers have reported negative correlations between time spent video-gaming and college students' GPA [5], and secondary students' school grades [6], [7], others have found no relationship between video-gaming and school grades [8], [9], [10]. Despite this limited empirical support, the suggestion that video-games may negatively affect academic performance has received widespread media attention (for example, [11]–[14]). A more comprehensive examination of the effect of video-gaming on academic performance is required.

Specifically, a number of systematic limitations in the extant literature make it difficult to assess the true relationship between academic performance and video-game use. First, most research has used school grades – which contain an inherent subjectivity on behalf of the assessor, and the effects of assessor expectations on student performance are well-documented (see, for example, [15]) – as outcome measures. A number of widely-cited studies have also relied on students' self-report assessments of their academic performance [7], [16]–[18]. These two indices of academic achievement are inherently subjective, and thus vulnerable to assessor subjectivity effects. For example, students' self-reports of their average grades may underestimate actual performance in accordance with perceived questionnaire demands [19], and teachers' preconceptions about students who play videogames may influence their subjective grading of students' performance (for example, [15]). Using standardized tests of academic performance negates these assessor subjectivity effects. Second, previous research has typically investigated the phenomena across few school sites, which increases the risk of sociocultural factors at particular school sites confounding the results. For example, in particular schools, students who play videogames may also be a peer-group who performs poorly academically, while in other schools the reverse may be true. Alternatively, across schools, the groups of students who play videogames may be more or less homogenous in terms of academic performance. Third, previous research has often used relatively small samples (e.g., 64 participants, [20]), reducing the reliability of findings.

In contrast to findings based on subjective indices of academic achievement, recent research examining the effects of violent videogames across 333 Hispanic youth revealed little-to-no relationship between a psychometrically valid measure of mathematics performance (the *Wide Range Achievement Test-IV*, [21]) and videogame exposure [22]. Thus, although *pathological* gaming has been consistently associated with poorer academic outcomes [16], [23]–[25], recent work indicates that this relationship may not hold for non-pathological game use [24], [25]. We follow on from this work, and avoid the aforementioned methodological issues, by investigating the relationship between video-gaming and psychometrically valid measures of academic performance in science, mathematics and reading across more than 192,000 students in 7,423 schools within 22 countries.

An additional, potentially important but relatively unexamined consideration is whether the effects of videogames on academic performance vary according to whether the games are played alone (single-player) or in collaboration with others (multiplayer). The social aspects of multiplayer games, together with their inherent reward structures, are intended to increase the games' appeal and the time people spend playing [26]. This increased playing time might result in additional displacement of homework and school related activities, leading to a greater decline in academic performance [4]. Consistent with this idea, participants randomly assigned to play multiplayer (cf. single player) games self-report greater interference in their sleep and academic work [27].

One concern with random assignment in videogame research is that, in natural settings, people who experience negative effects from gaming can choose not to play, or may have their gaming behavior curtailed. While research with small samples suggests that boys randomly assigned to be given a new videogame console initially perform poorer academically compared to those who have never owned a games console [20], regular gamers may habituate to the activity, attenuating negative effects on everyday functioning. Further, although providing new videogame consoles to randomly selected children may result in initial declines in academic performance (cf. children who do not possess game consoles), without the appropriate control, it is not clear if this effect reflects properties inherent to gaming behavior or a more general displacement mechanism attributable to the opportunity to engage in a novel activity. If the latter is true, then it might be expected that in an older cohort who have had the opportunity to engage in playing videogames for a greater period of time, the relationship between videogames and academic performance may be weaker or even negligible.

We reanalyzed one of the largest educational datasets ever produced to estimate the true effect size of video-gaming on academic performance among adolescent gamers, and test for differences between single player and multiplayer gamers.

## Method

We reanalyzed data from over 192,000 students (aged ∼15 years) across 22 OECD countries assessing the frequency of single- and multiplayer video-gaming (never/hardly ever, once/twice a month, once/twice a week, daily), and including standardized psychometric measures of performance in science, mathematics and reading ability [28]. We present effect sizes in the absence of hypothesis tests as the large number of participants greatly increases the risk of Type-I error.

As video-gaming is most prevalent in Western industrialized nations, we used three criteria for inclusion in the analyses. To ensure that the country was both Western and industrialised, the country had to be an OECD nation, and be classified by the International Monetary Fund as an advanced economy [29] to be included. Second, the country had to have data on the frequency of video-gaming in the PISA dataset [28]. These exclusion criteria left 23 countries. Finally, we excluded South Korea from the analysis due to their non-representatively high prevalence of video-gaming (associated with the rise of e-sports and video-gaming culture). Indeed, the prevalence rate of video-game addiction in South Korea is estimated to be more than double that of any other country [30]. Although data from South Korea were excluded from our primary analyses, we present results including the South Korean data in Figure S1 in the supplementary materials (available online). Within the remaining countries, 192,975 students indicated their frequency of single-player videogame use and 192,741 students indicated their frequency of multiplayer videogame use.

Science, mathematics and reading ability were all assessed on a scale with an international average of approximately 500 and a standard deviation of approximately 100. For reading assessments, students read a section of text and then answered comprehension questions (e.g., interpreting, summarizing, or applying the information contained within the text). For mathematics, students engaged in mathematical calculation and interpretation (e.g., calculating the area of objects or accurately interpreting graphs). For science, students applied scientific thinking (e.g., interpreting the results of scientific experiments, deciding upon the best design for potential experiments, and determining causal factors in particular scenarios) [31]. A complete list of sample questions can be obtained from the OECD [31].

The 2009 PISA dataset contains five sets of plausible values for each of these constructs. These values represent Rasch model estimates of student performance based upon the differences in test version and, thus, a range of plausible performance for each student. In accordance with OECD [32] recommendations, we analysed all five plausible values separately and present average performance across these analyses. One plausible value for mathematics and science in the analyses of multiplayer gaming failed to converge, as well as one plausible value for mathematics in the analyses of single player videogame use. For these analyses the results were averaged across the remaining four plausible values.

To examine the relationship between videogame use and academic performance, we first recoded the frequency of single player videogame use into three dummy variables: single player daily (0, no; 1 yes), single player weekly (0, no; 1 yes) and single player monthly (0, no; 1 yes). Thus, someone who never played video games scored three zeros, while a student who played daily scored two zeros and a 1. The same dummy coding was undertaken for the frequency of multiplayer videogame use. Data were analysed using multilevel models and the iterative generalized least squares (IGLS) method [33]. Within each multilevel model, academic performance was nested within one first level variable (school site) and one second level variable (country). Intercepts and slopes were allowed to vary across each level of the data. Thus, each school was allowed to have a unique intercept and slope within the country's average, and each country was allowed a unique intercept and slope. The multilevel models allowed the relationship between videogame use and academic performance to vary across countries, and between school sites. The models are described mathematically by equations 1 and 2.
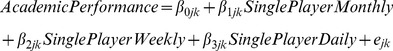
(1)

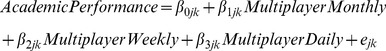
(2)Where Academic Performance was the plausible value to be analysed (e.g., PV1Science), *e* represents the residual error term, *j* represents the value was allowed to vary by school site, and *k* represents that the value was allowed to vary by country.

## Results

**Figure 1:**
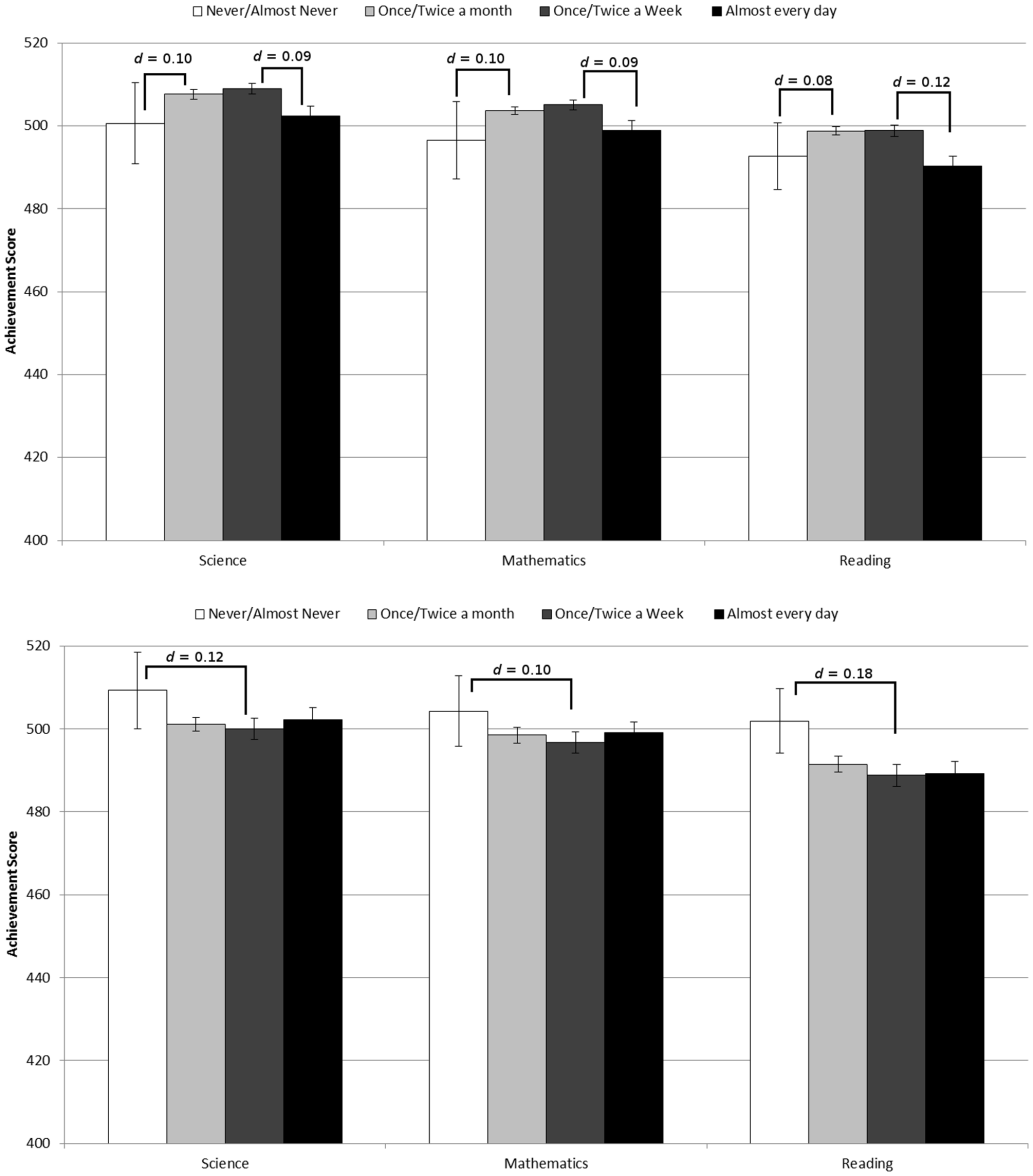
Frequency of single-player (top) and multiplayer (bottom) game use and science and mathematics performance. Error bars represent 95% confidence intervals. As MLwiN does not calculate confidence intervals for multi-level models, we estimated confidence intervals as 1.96 times the standard error of the multilevel model slopes, as recommended in the MlwiN Manual [34].

Multilevel models allowed the relationship between videogame use and academic performance to vary across countries and schools to obtain the best estimate of the effect of video-gaming on academic achievement [32]. Results are displayed in Figure 1. As can be seen in the figure, there is no evidence that academic performance in science, mathematics or reading ability, declined as a function of increased gameplay frequency, for single player or multiplayer videogame use. Most differences in student performance across video-gaming frequencies were negligible (*ds*<0.2). The largest performance *decline* associated with increased video-gaming was in reading, with a difference that approached, but did not exceed the cut off for a small effect (*d* = 0.18) between students playing multiplayer games daily and those that never played. All other effects were well below the guidelines for a small effect (*d*s<0.2, see Figure 1). While the findings relating to mathematical performance support prior research [22], the negligible and non-existent declines in reading and science associated with increased videogame use contradict previous suggestions that videogames are generally detrimental to academic performance (cf. [22]). Results including South Korea were qualitatively similar to the results presented here. Note that as *ds* were calculated using residual variance after accounting for between country and school variances, they potentially overestimate the relationship between video-gaming and academic performance.

Table 1 shows the standard deviations for the difference between frequency of videogame play across countries and schools. As can be seen, despite some variance, the results are relatively consistent across countries and school sites.

**Table 1:** Standard deviations for the relationship between frequency of videogame use and academic performance for single player and multiplayer videogames across countries and schools.

	Single player gameplay frequency				Multiplayer gameplay frequency			
Variance across countries	*Never*	*Monthly*	*Weekly*	*Daily*	*Never*	*Monthly*	*Weekly*	*Daily*
*Science*	23.38	1.46	2.25	5.07	21.97	3.45	5.46	6.10
*Mathematics*	22.00	0.00	2.05	4.95	20.28	3.86	5.27	5.57
*Reading*	18.94	1.02	2.46	4.93	18.37	3.81	5.46	6.48
**Variance Across Schools**								
*Science*	54.92	4.03	8.39	18.08	53.57	3.91	10.41	17.35
*Mathematics*	55.55	6.50	8.19	16.05	54.53	4.86	11.30	15.87
*Reading*	57.48	5.47	8.59	18.31	55.07	3.53	10.38	18.02

## Discussion

We examined the effect of video-gaming on academic performance in an ecologically valid data set, using standardized assessments of academic performance for participants who self-select to engage in video-gaming behavior. Generally, we found little association between video-gaming frequency and academic performance. These data seriously challenge general claims that academic performance is negatively related to the frequency of videogame play (e.g., [4]).

One explanation for the discrepancy between our results and previous findings may be that PISA's psychometrically valid standardized tests attenuate assessor subjectivity effects inherent in teacher reports and self-reports of school grades (often used as outcome measures in previous research). Research demonstrates that teachers' assessments of student performance are inherently subjective, and are vulnerable to non-veridical influences, including judgments of the student's attitudes and hobbies [15]. Self-reports are similarly vulnerable to non-veridical influences [19]. Another possibility is that those who find that video-games interfere with their schooling may choose not to play or to reduce time spent playing, or have this choice made for them (e.g., by parents). Alternatively, regular gamers may habituate to the activity, attenuating negative effects on academic outcomes.

An advantage of using multilevel modelling analyses is that the relationship between academic performance and video-gaming is allowed to statistically vary across school sites and countries. Within the present data, there was relatively little variance in the relationship between videogame use and academic performance across countries and schools. However, the fact that some variance occurred, particularly at the school level, suggests that increased video-gaming was associated with reductions in academic performance in some schools, but increased performance in others. Thus, with a view to future research, focusing upon any one school is unlikely to provide a good understanding of video-gaming effects on academic achievement. Our results support the need for psychological and educational researchers to examine effects across appropriately large and diverse datasets to ensure their validity.

In sum, across more than 192,000 students in 22 countries, video-gaming behaviour had little effect on psychometrically valid assessments of academic performance in science, mathematics, or reading. The results suggest that the impact of video-gaming on academic performance is too small to be considered problematic.

## Supporting Information

Figure S1 **Frequency of single-player (top) and multiplayer (bottom) game use and science and mathematics performance including South Korea.** Error bars represent 95% confidence intervals. As MLwiN does not calculate confidence intervals for multi-level models, we estimated confidence intervals as 1.96 times the standard error of the multilevel model slopes, as recommended in the MlwiN Manual [34].
(TIFF)
