# Supplementary figures and images for: Video-Games Do Not Negatively Impact Adolescent Academic Performance in Science, Mathematics or Reading
Source: PLoS One. 2014 Apr 3;9(4):e87943. doi: 10.1371/journal.pone.0087943 (PMC3974676; doi:10.1371/journal.pone.0087943)

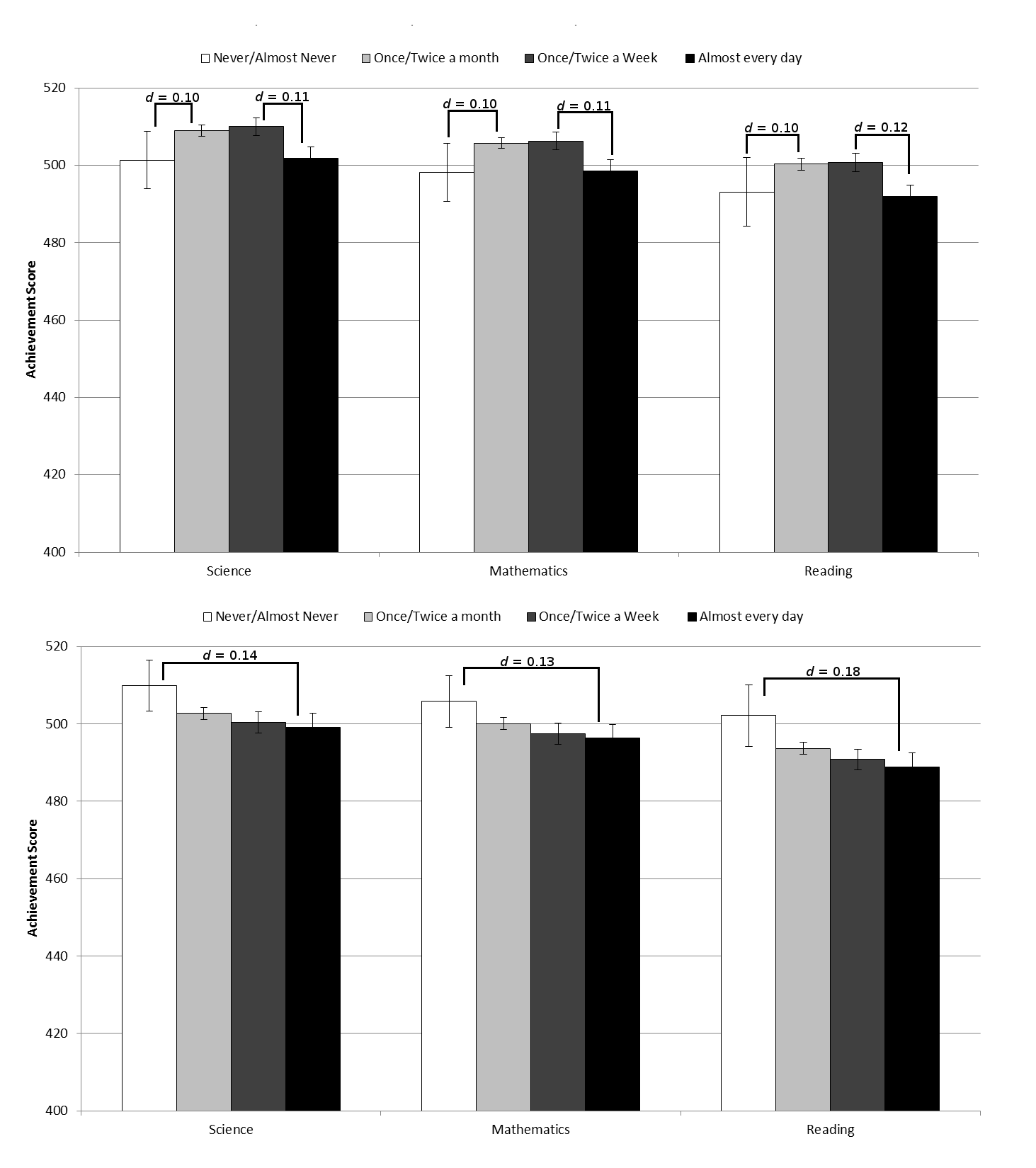

Supplement: Figure S1 — Frequency of single-player (top) and multiplayer (bottom) game use and science and mathematics performance including South Korea. Error bars represent 95% confidence intervals. As MLwiN does not calculate confidence intervals for multi-level models, we estimated confidence intervals as 1.96 times the standard error of the multilevel model slopes, as recommended in the MlwiN Manual [34]. (TIFF) [file pone.0087943.s001.tif]
